# Identification and characterization of the *RcTCP* gene family and its expression in response to abiotic stresses in castor bean

**DOI:** 10.1186/s12864-024-10347-6

**Published:** 2024-07-04

**Authors:** Yanxiao Li, Xingyang Liu, Xingyuan Xu, Guishuang Zhu, Dianjun Xiang, Peng Liu

**Affiliations:** College of Agriculture Life Science, Inner Mongolia Minzu University, Tongliao, 028000 China

**Keywords:** Abiotic stress, Castor bean, Subcellular localization, *TCP* gene family

## Abstract

**Background:**

The *TCP* (*teosinte branched1/cincinnata/proliferating cell factor*) family plays a prominent role in plant development and stress responses. However, *TCP* family genes have thus far not been identified in castor bean, and therefore an understanding of the expression and functional aspects of castor bean *TCP* genes is lacking. To identify the potential biological functions of castor bean (*RcTCP*) *TCP* members, the composition of *RcTCP* family members, their basic physicochemical properties, subcellular localizations, interacting proteins, miRNA target sites, and gene expression patterns under stress were assessed.

**Results:**

The presence of 20 *RcTCP* genes on the nine chromosomes of castor bean was identified, all of which possess TCP domains. Phylogenetic analysis indicated a close relationship between *RcTCP* genes and *Arabidopsis AtTCP* genes, suggesting potential functional similarity. Subcellular localization experiments confirmed that RcTC01/02/03/10/16/18 are all localized in the nucleus. Protein interaction analysis revealed that the interaction quantity of RcTCP03/06/11 proteins is the highest, indicating a cascade response in the functional genes. Furthermore, it was found that the promoter region of *RcTCP* genes contains a large number of stress-responsive elements and hormone-induced elements, indicating a potential link between *RcTCP* genes and stress response functions. qRT-PCR showed that all *RcTCP* genes exhibit a distinct tissue-specific expression pattern and their expression is induced by abiotic stress (including low temperature, abscisic acid, drought, and high salt). Among them, *RcTCP01/03/04/08/09/10/14/15/18/19* genes may be excellent stress-responsive genes.

**Conclusion:**

We discovered that *RcTCP* genes play a crucial role in various activities, including growth and development, the stress response, and transcription. This study provides a basis for studying the function of *RcTCP* gene in castor.

**Supplementary Information:**

The online version contains supplementary material available at 10.1186/s12864-024-10347-6.

## Background

Castor bean (*Ricinus communis*) is an important oil crop that has been used as a model plant for improving saline-alkali soil in recent years [1]. In recent years, with the occurrence of extreme weather globally, the first affected are the plants [2]. As a sessile organism, plants find it difficult to avoid environmental damage, therefore they have evolved complex protective mechanisms, including the regulation of stress response genes by recognizing adverse signals and regulating gene expression and transcription [3, 4]. Therefore, identifying and exploring the function of plant stress-regulating genes is of great significance for studying the protective mechanisms of plants in adapting to stressful environments.

The *TCP* (t*eosinte branched1/cincinnata/proliferating cell factor*) gene, as a plant-specific transcription factor, plays an important role in plant growth, development, and the response to abiotic stress [5]. The name *TCP* originates from the initial discovery of several genes, including the *TB1* (*teosinte branched* 1) gene from maize (*Zea mays*), the *CYC* (*bycloidea*) gene from snapdragon (*Antirrhinum majus*), and the *PCF1*/*2* (*proliferating cell factors 1/2*) genes from rice (*Oryza sativa*) [6]. These genes all have a non-canonical helix–loop–helix (bHLH) domain consisting of 59 amino acid residues, namely the TCP domain [7]. Based on the differences in the TCP domain, previous researchers classified *TCP* gene family members into two classes: Class I and Class II [8]. Class I lacks four amino acid residues in the domain region compared to Class II [9], which is further divided into two sub-branches, CIN and CYC/TB1, based on protein sequence differences [10].

Research has found that *TCP* family genes mainly regulate the growth, development, and physiological and biochemical responses of plants in the meristematic tissues, such as the development of vascular tissue [11], seed germination [12], and the circadian rhythm [13]. In addition, *TCP* genes can increase plant tolerance to stress by regulating cell osmotic pressure or changing cell permeability [14–16]. For example, the *OsNHX1* gene in rice, as a key gene regulating cell permeability, can bind specifically to *OsPCF2* to activate the expression of the *OsNHX1* gene and thus enhance salt tolerance [14]. Heterologous expression of the *OsTCP19* gene effectively reduces water loss and the accumulation of reactive oxygen species in *Arabidopsis*, increasing the resistance of both seedlings and mature plants [16]. Overexpression of the *OsTCP14* and *OsTCP21* genes decreases the tolerance of transgenic rice to low temperature stress, while Osa-miR319b targets and regulates the *OsTCP14* and *OsTCP21* genes and reduces the cold tolerance of rice, indicating the involvement of *TCP* genes at the post-transcriptional level [17]. Furthermore, Osa-miR319a also negatively targets the *OsTCP21* gene to regulate tiller number in transgenic rice, thereby reducing yield [17]. Furthermore, the *TCP* gene plays a significant role in controlling plant cell proliferation and leaf differentiation [18, 19]. For instance, overexpression of the *LcTCP4* gene in *Arabidopsis* leads to transgenic plants exhibiting a smooth leaf margin phenotype [18]. The *StAST1* gene in potatoes inhibits tuber proliferation by mediating plant hormone signal transduction [19].

Research indicates that redundant functionality exists in TCP proteins of the same species [20]. For example, in *Arabidopsis thaliana*, *AtTCP5* and *AtTCP17* can both bind and transcribe the *PIF4* gene and also interact with the *PIF4* gene to enhance the transcriptional activity of the downstream genes *YUC8* and *IAAI19* [21, 22]. In banana (*Musa paradisiaca*), *MaTCP20* can form dimers with *MaTCP5* and *MaTCP19*, regulating the transcription level of *XTH10*/*XTH11* genes to affect plant stress resistance [23]. In *Phalaenopsis equestris*, *PePCF10* and *PeCIN8* can form homodimers, regulating ovule development in floral tissues [24].

Currently, the online website PlantTFDB (http://planttfdb.gao-lab.org/) has predicted the transcription factor members and their numbers for plants with completely sequenced genomes [25]. The number of *TCP* genes totals 4,187, with significant variations in the number of *TCP* gene family members across different species. Tobacco (*Nicotiana tabacum*), cultivated soybean (*Glycine max*), and *Brassica napus* var. *pekinensis* have the largest numbers of *TCP* genes, with 96, 81, and 76 genes, respectively [25]. However, castor bean TCP transcription factor family has not been identified. Thus, identifying and understanding the *TCP* gene in castor bean and its potential biological functions is of great significance for breeding superior castor bean varieties with resistance. In this study, we used a bioinformatics approach to screen and identify the castor bean *TCP* gene family. Comprehensive analysis was conducted on the chromosomal localization, physicochemical properties, potential biological functions, subcellular localization, and gene expression of these genes. The results inform a comprehensive understanding of the biological functions of the castor bean *TCP* gene family throughout growth and development.

## Results

### Identification and basic information of castor *RcTCP* genes

A total of 20 members of the *TCP* gene family were identified from the entire genome of castor bean, as revealed in Table 1. The length of the proteins encoded by these genes varied, ranging from 185 amino acids for RcTCP19 to 567 amino acids for RcTCP12. Their molecular weights also varied, ranging from 20246.68 kDa for RcTCP19 to 63947.78 kDa for RcTCP12. The theoretical isoelectric points ranged from 6.36 for RcTCP17 to 9.57 for RcTCP11. The instability index of the proteins encoded by *RcTCP* genes ranged from 38.45 for *RcTCP11* to 67.87 for *RcTCP05*. The aliphatic index ranged from 53.61 for RcTCP15 to 90.02 for RcTCP20. Notably, the average hydrophilicity value of these proteins was less than 0, indicating their hydrophilic nature. Among them, RcTCP20 (− 0.115) exhibited the strongest hydrophilicity, while RcTCP07 (− 0.883) had relatively weaker hydrophilicity. Furthermore, the subcellular localization prediction results of the RcTCP proteins indicated that, 20 RcTCP proteins were all located in the nucleus,

**Table 1:** Analysis of the basic information of the *RcTCP* gene and its encoded protein

Gene name	Gene ID	Number of amino acid	Molecular weight (Da)	Theoretical PI	Instability index	Aliphatic index	Grand average of hydropathicity	Subcellular location
*RcTCP01*	Rc01T000220.1	341	36151.18	9.46	59.12	73.61	-0.254	Nucl.
*RcTCP02*	Rc01T000421.1	417	47460.85	7.64	53.53	60.86	-0.858	Nucl.
*RcTCP03*	Rc01T000636.1	420	45511.23	7.95	63.22	62.26	-0.684	Nucl.
*RcTCP04*	Rc02T003729.1	441	46812.56	6.90	50.02	64.24	-0.597	Nucl.
*RcTCP05*	Rc04T008105.1	401	43156.44	7.98	67.87	58.48	-0.741	Nucl.
*RcTCP06*	Rc04T008233.1	371	41211.65	7.86	42.53	63.91	-0.696	Nucl.
*RcTCP07*	Rc04T008382.1	449	50158.55	8.78	45.14	54.12	-0.883	Nucl.
*RcTCP08*	Rc05T010862.1	497	53900.99	7.08	47.99	58.19	-0.820	Nucl.
*RcTCP09*	Rc05T011021.1	394	43846.59	9.44	53.2	75.81	-0.588	Nucl.
*RcTCP10*	Rc05T011793.1	377	41976.50	7.27	44.75	63.13	-0.819	Nucl.
*RcTCP11*	Rc06T014031.1	229	24209.89	9.57	38.45	69.08	-0.525	Nucl.
*RcTCP12*	Rc06T014210.1	567	63947.78	8.43	57.45	65.41	-0.835	Nucl.
*RcTCP13*	Rc07T015145.1	197	21719.62	7.85	49.65	62.44	-0.536	Nucl.
*RcTCP14*	Rc07T017187.1	344	37407.57	9.04	53.56	64.42	-0.714	Nucl.
*RcTCP15*	Rc08T017972.1	546	56768.21	7.28	55.46	53.61	-0.656	Nucl.
*RcTCP16*	Rc09T019649.1	302	32604.44	9.37	49.38	61.46	-0.676	Nucl.
*RcTCP17*	Rc09T021856.1	409	45866.57	6.36	57.92	62.54	-0.830	Nucl.
*RcTCP18*	Rc09T021857.1	424	47255.92	7.01	45.38	59.43	-0.855	Nucl.
*RcTCP19*	Rc10T022169.1	185	20246.68	7.14	66.18	69.78	-0.672	Nucl.
*RcTCP20*	Rc10T022413.1	442	48146.35	6.42	47.61	90.02	-0.115	Nucl.

### Analysis of *RcTCP* gene structure and conserved motifs and domains of the encoded proteins

**Fig. 1:**
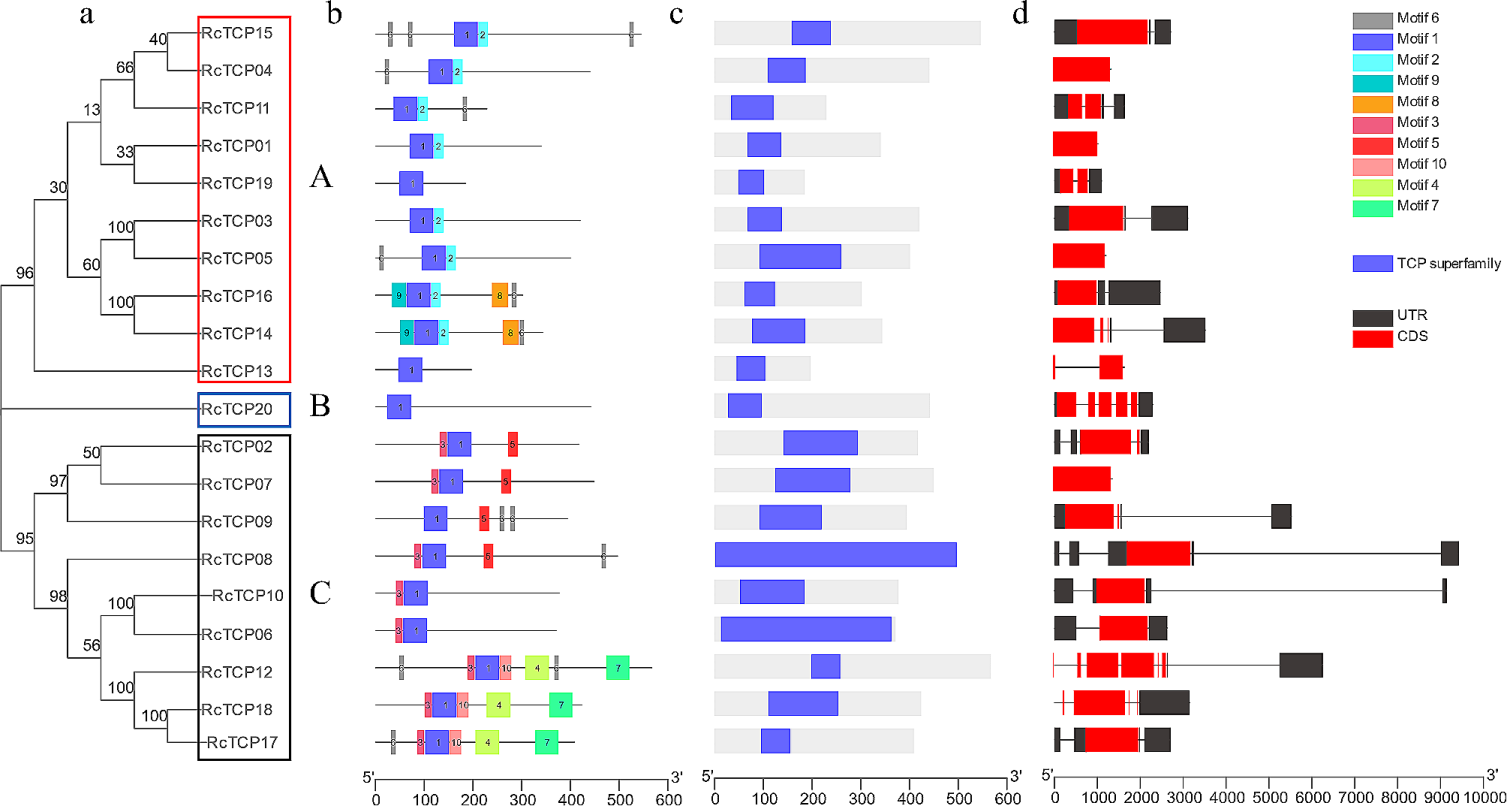
Phylogenetic relationships, conserved protein motifs, domains, and gene structures of RcTCP genes. (**a**) Phylogenetic tree of 20 RcTCP proteins. (**b**) Motif composition analysis of castor RcTCP proteins indicated 10 conserved motifs. (**c**) The domain of RcTCP genes. (**d**) *RcTCP* gene structure. Black lines indicate introns; the red box represents the CDS; the black box represents the UTR

In Fig. 1, the 20 RcTCP proteins were categorized into three groups, namely A, B, and C (Fig. 1a). The motif composition was similar within each group. In group A, all members except RcTCP13 lacked Motif 2. In group C, all members except RcTCP09 lacked Motif 3 (Fig. 1b). Additionally, all 20 RcTCP proteins contain a conserved TCP superfamily domain at the N-terminus (Fig. 1c), indicating accurate identification. Furthermore, all RcTCP proteins possessed a conserved Motif 1 (Fig. 1b). Further analysis revealed that Motif 1 was situated within the TCP superfamily domain, suggesting its important role in RcTCP proteins (Fig. 1c). Moreover, the gene structure of *RcTCP* varied, with 1–7 exons and 0–4 introns detected (Fig. 1d). However, based on the evolutionary system analysis, genes within the same group exhibit similarity in gene structure, such as *RcTCP01*, *RcTCP03*, and *RcTCP04* of group A (Fig. 1d).

### The position and collinear relationship of castor *RcTCP* genes in the chromosomes

**Fig. 2:**
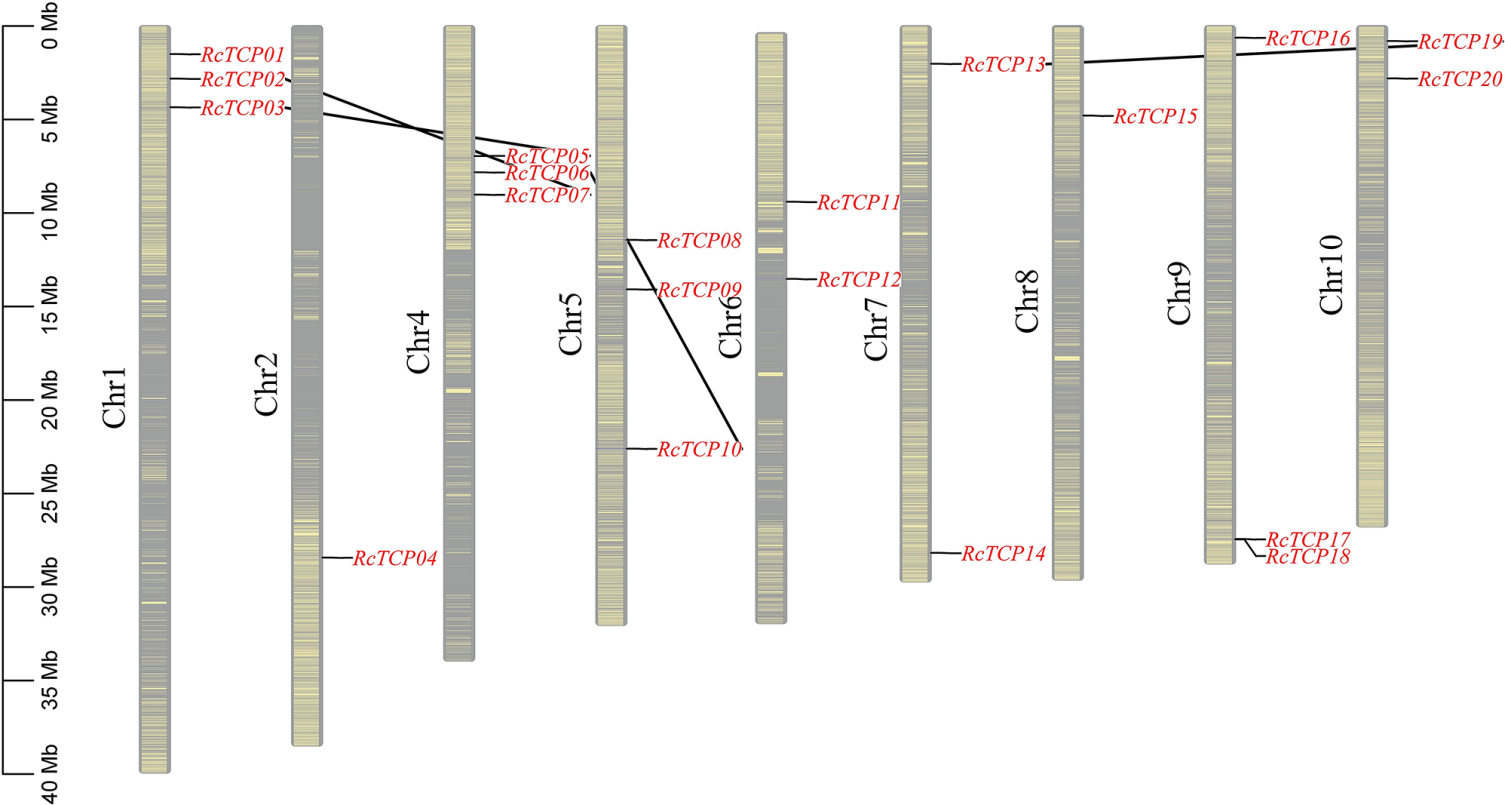
Position and collinear relationship of castor *RcTCP* genes in the chromosomes. The red lines indicate collinearity between genes

As depicted in Fig. 2, the *RcTCP* genes were distributed across all 10 chromosomes of castor bean. Among them, chromosomes 2 and 8 exhibited the lowest number of *RcTCP* genes, with one gene each, namely *RcTCP04* and *RcTCP15*. In addition, out of the 20 genes, four pairs of genes displayed syntenic relationships: *RcTCP02*-*RcTCP07*, *RcTCP03*-*RcTCP05*, *RcTCP06*-*RcTCP10*, and *RcTCP13*-*RcTCP19*. Furthermore, the Ka/Ks values for these homologous gene pairs were all less than 1 (Table S2). This indicates that the *RcTCP* gene has undergone negative purifying selection in the process of evolution, and there may be functional similarity between genes.

### Prediction of the secondary and tertiary structure of RcTCP proteins

**Fig. 3:**
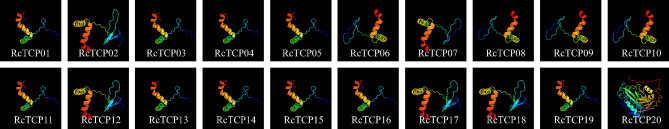
Predicting the tertiary structure of RcTCP proteins

The analysis of the secondary and tertiary structures of RcTCP proteins can provide some basic materials for the follow-up study of protein function. The secondary structure of the RcTCP proteins indicated that alpha-helixes accounted for 15.00–37.84%, extended strands accounted for 8.80–21.41%, and random coils accounted for 44.42–72.08%, without β-turn structures (Table S3). The random coil structure was the most abundant in the tertiary structure of the RcTCP proteins, consistent with the secondary structure prediction results (Fig. 3).

### Phylogenetic evolution, collinearity, and selection pressure analysis of the *TCP* gene family

**Fig. 4:**
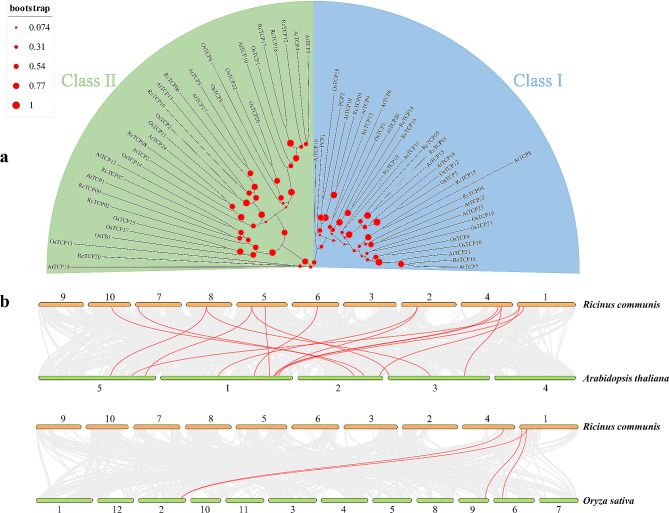
Inter-species evolutionary tree and collinearity analysis of TCP proteins. (**a**) Inter-species evolutionary tree analysis; Rc, Os, and At represent castor bean, rice, and *Arabidopsis thaliana* TCP proteins. (**b**) Inter-species collinearity analysis; red lines indicate collinear genes

Castor bean, *Arabidopsis*, and rice had a total of 66 TCP proteins, which were divided into two groups, Class I and Class II. There were 33 TCP proteins in each group, with 10, 11, and 12 Class II TCP proteins in castor bean, *Arabidopsis*, and rice (*Oryza sativa*), respectively, and 10, 13, and 10 Class I TCP proteins in castor bean, *Arabidopsis*, and rice (*Oryza sativa*), respectively, with similar distributions (Fig. 4a). Interestingly, 11 *RcTCP* genes in castor bean had 16 collinear relationships with 15 genes in *Arabidopsis*, and three *RcTCP* genes had four collinear relationships with three *OsTCP* genes in rice, indicating that these genes are paralogous (Fig. 4b). To better understand the evolutionary constraints on the *TCP* gene family, selection pressure analysis was conducted on these paralogous gene pairs (Table S2). The *RcTCP* genes in castor exhibited a distant evolutionary distance from eight and four *TCP* genes in *Arabidopsis* and rice, and the Ks values could not be calculated. These gene pairs, including *RcTCP18*-*AtTCP10*, *RcTCP04*-*AtTCP22*, *RcTCP01*-*AtTCP9*, *RcTCP01*-*AtTCP19*, *RcTCP14*-*AtTCP6*, *RcTCP19*-*AtTCP11*, *RcTCP10*-*AtTCP13*, *RcTCP10*-*AtTCP5*, *RcTCP05*-*OsTCP6*, *RcTCP03*-*OsTCP6*, *RcTCP03*-*OsTCP12*, and *RcTCP01*-*OsTCP18*, have extensive sequence divergence, with most of the potentially synonymous mutation sites experiencing synonymous mutations. It is hypothesized that orthologous genes in different plants may exhibit functional similarities.

### Prediction of the *cis*-acting elements of *RcTCP* gene promoters in castor

**Fig. 5:**
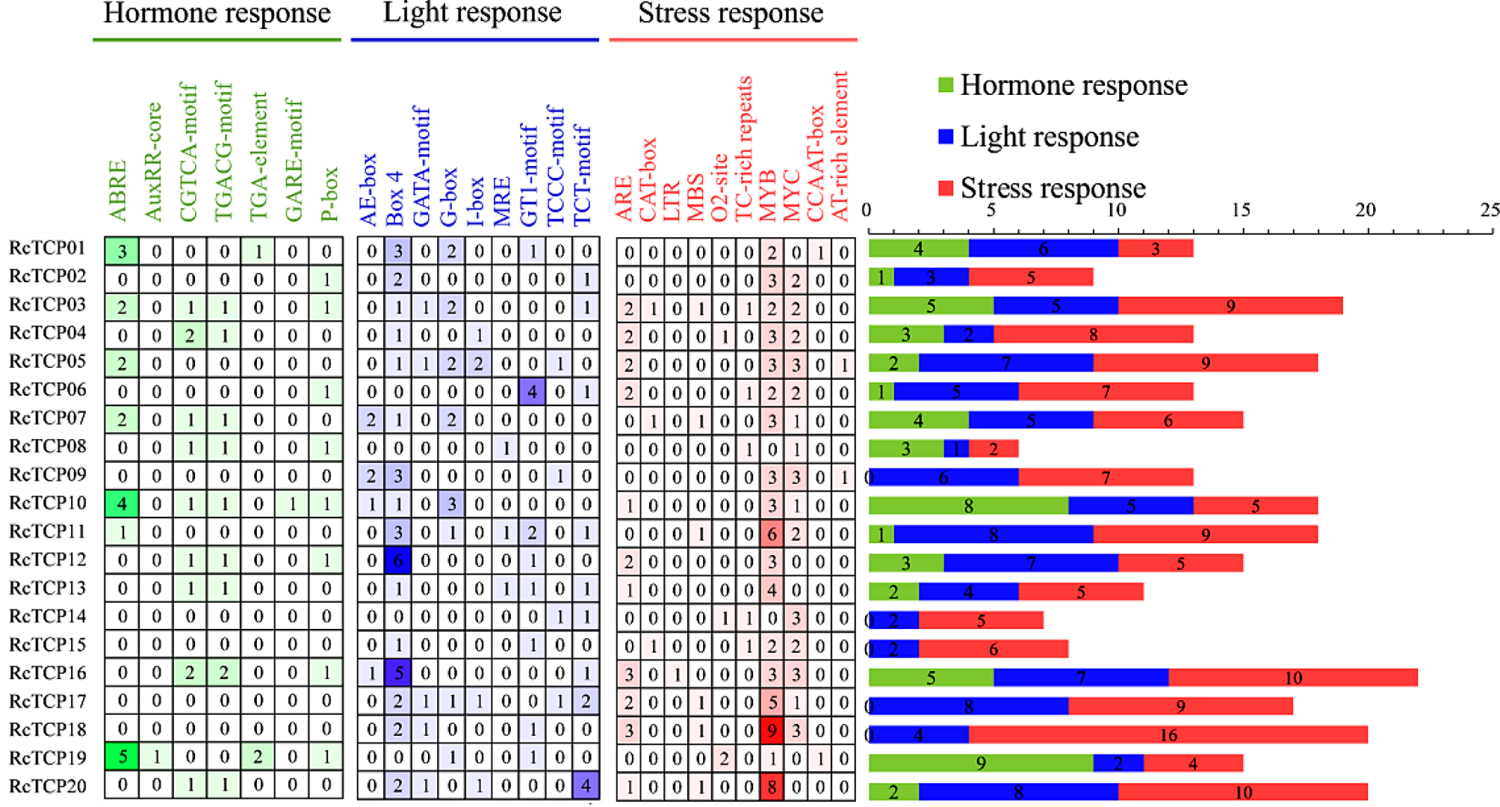
Prediction of the cis-acting elements of the RcTCP promoter. The numbers in the figure represent the quantity of elements

According to the diagram in Fig. 5, the promoter region of the *RcTCP* gene contained a total of 26 *cis*-acting elements, including seven hormone-response elements, nine light-response elements, and 10 stress-response elements, totaling 290 elements. Among the hormone-response elements, ABRE was the most abundant, accounting for 35.85% of the total, and was mainly distributed in the promoters of the *RcTCP10* and *RcTCP19* genes. Among the light-response elements, Box 4 was the most abundant, accounting for 36.08% of the total, and was mainly distributed in the promoters of the *RcTCP12* and *RcTCP16* genes. Among the stress-response elements, MYB and MYC elements were relatively abundant, accounting for 46.43% and 22.14% of the total number of elements, respectively. Notably, all *RcTCP* gene promoters contained MYB elements except *RcTCP08* and *RcTCP14* gene. Among them, the promoters of the *RcTCP20*, *RcTCP18*, and *RcTCP11* genes had a higher number of MYB elements, with nine, eight, and six elements, respectively. Based on these results, we speculate that *RcTCP* genes may be involved in plant responses to stress through the binding of MYB or MYC elements.

### Subcellular localization analysis of RcTCP proteins

**Fig. 6:**
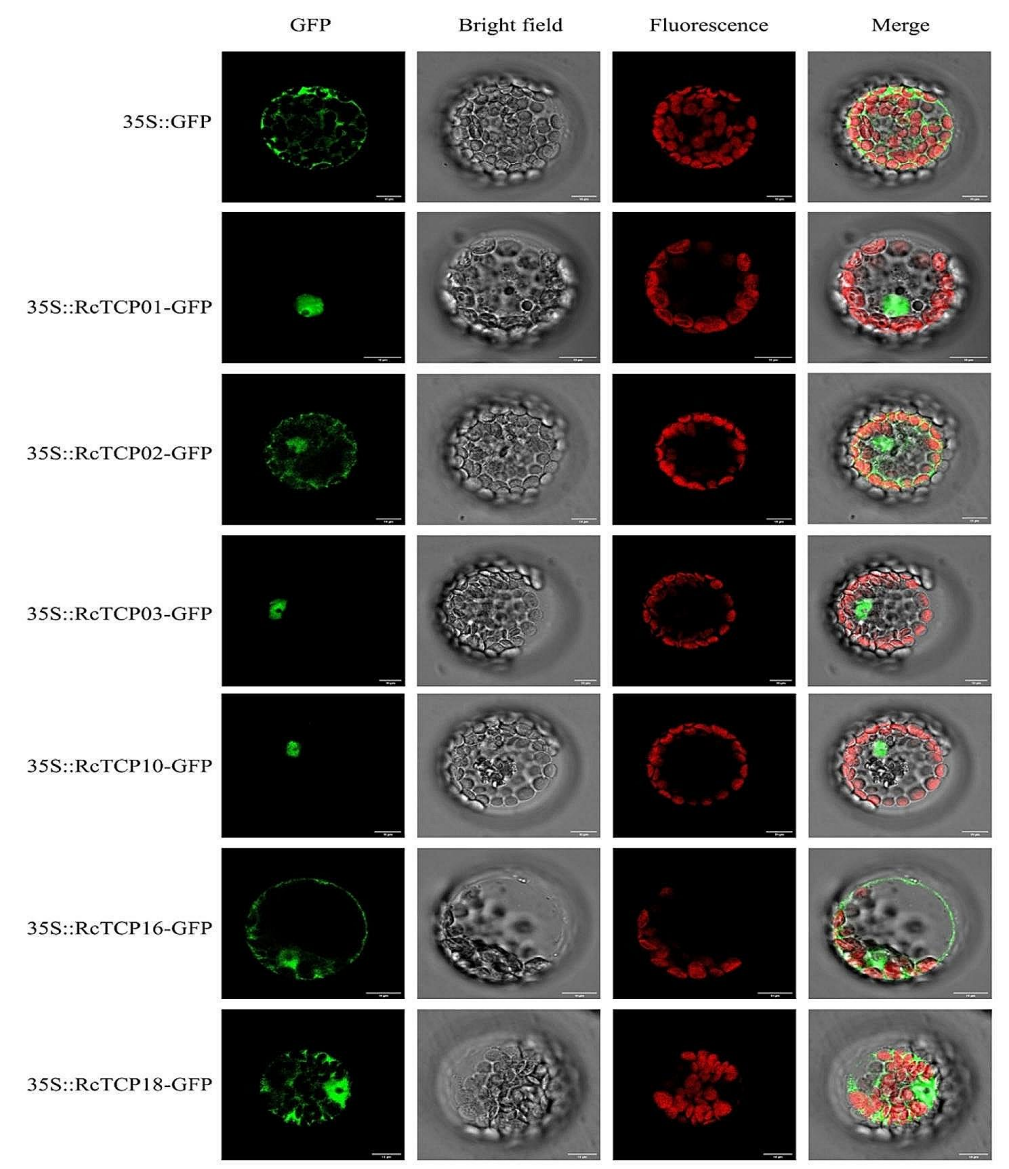
Subcellular localization of RcTC01/02/03/10/16/18 proteins. *Note*: GFP indicates the green fluorescence field, fluorescence stands for the chloroplast autofluorescence field, and Merge stands for the superimposed field. Excitation light wavelengths: GFP field: 488 nm, fluorescence field: 488 nm. *Note*: the green fluorescence and chloroplast autofluorescence excitation light wavelengths were the same, and the acquisition light wavelengths were different. The fluorescent images were observed using a confocal laser scanning microscope. Scale bar = 10 μm

To clarify the subcellular location of RcTCP proteins, we constructed recombinant plasmids for genes *RcTC01*/*02/03*/*10*/*16*/*18*, with 35 S::GFP as a control, and transformed them into *Arabidopsis* protoplasts. The green fluorescence of 35 S::GFP was present in the cell nucleus, cell membrane, and cytoplasm, while the green fluorescence of 35 S::RcTC01/02/03/10/16/18-GFP was mainly distributed in the cell nucleus of *Arabidopsis* protoplast cells (Fig. 6). This indicates that the previously predicted subcellular location of the RcTCP proteins was consistent and reliable.

### Construction of the PPIs network of RcTCP and prediction of miRNA target sites on encoding genes

**Fig. 7:**
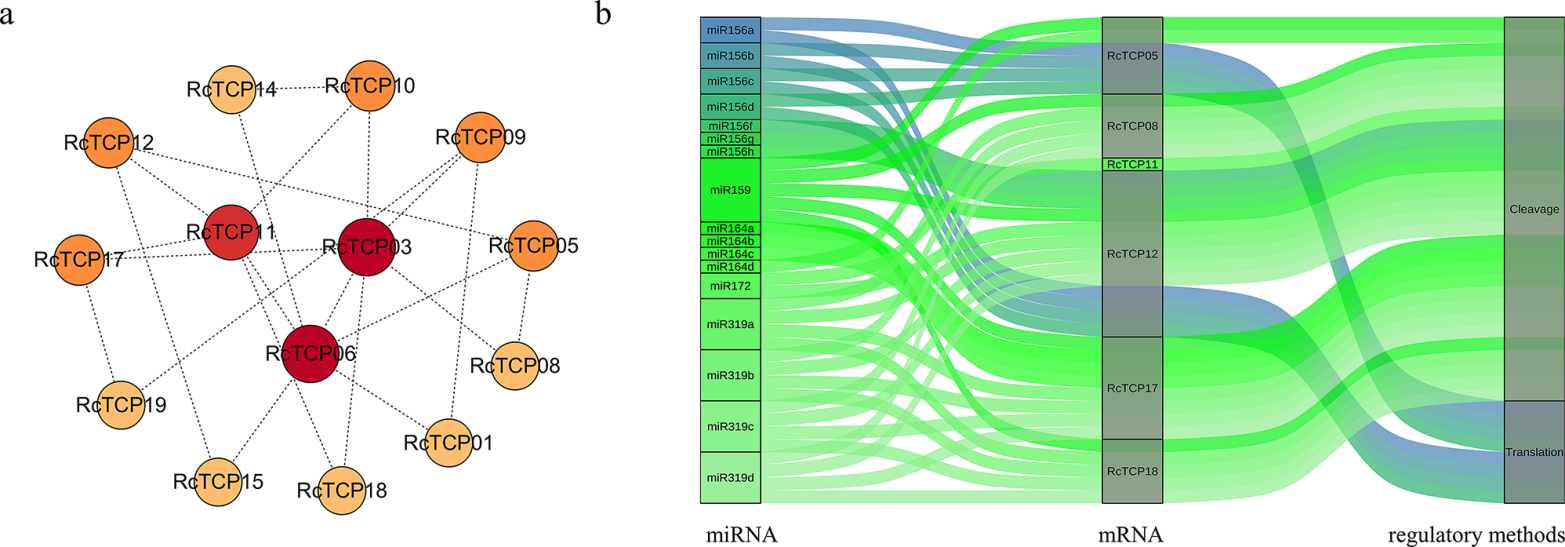
**(a)** Analysis of protein-protein interaction network of RcTCP. The darker the color, the higher the node degree. **(b)** Prediction of miRNA targets for the *RcTCP* gene

The PPI results (Fig. 7a) indicated that, apart from the lack of interaction between the RcTCP20 protein and other RcTCP proteins, the remaining 19 RcTCP proteins interacted. Among them, the proteins with the most interactions included RcTCP03, RcTCP06, and RcTCP11, with six, six, and five interacting proteins, respectively. This suggests that these interacting proteins may collectively participate in the regulation of growth and development as well as the stress response in castor plants.

miRNAs can interact with non-coding RNAs to form ceRNA networks that participate in regulating plant physiological activities. Therefore, the miRNAs targeted by *RcTCP* were predicted. Among the 20 *RcTCP* genes, six genes were predicted to have 17 miRNAs as targets, and 38 regulatory pathways were constructed (Fig. 7b). Among them, *RcTCP12* and *RcTCP17* had the most miRNA target sites, with 13 and eight, respectively. In addition, miR159 could regulate five target genes *RcTCP05*/*08/12*/*17*/*18* through cleavage. These results imply that *RcTCP* genes are expressed during various physiological activities in castor.

### Analysis of the tissue expression patterns of the *RcTCP* genes

**Fig. 8:**
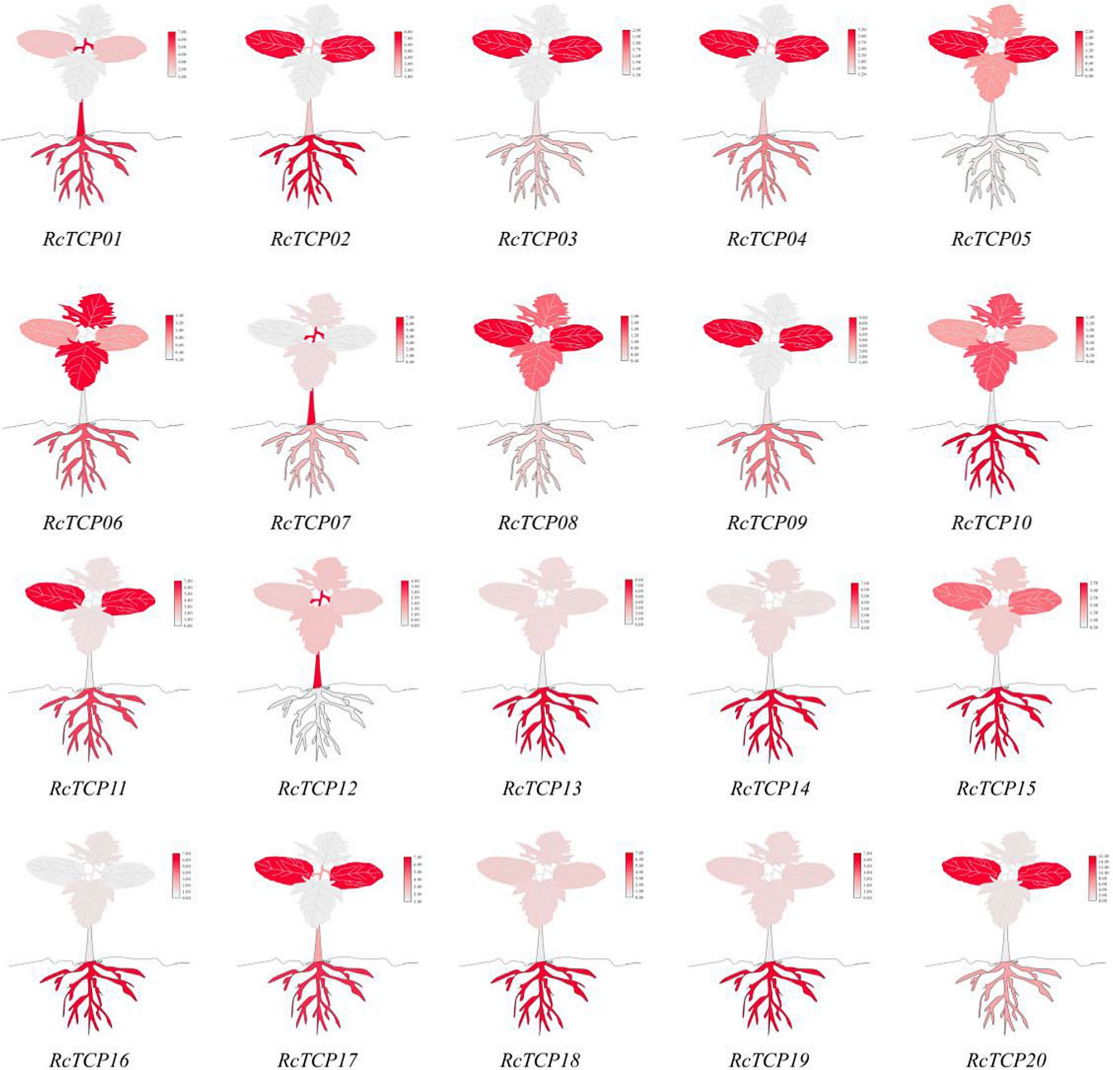
Analysis of the tissue-specific expression pattern of *RcTCP* genes. From bottom to top are the root, stem, cotyledon, and true leaf tissues of castor. Darker colors indicate higher relative expression levels of the gene. The expression values are the average of three biological replicates relative to the true leaf tissue control

As shown in Fig. 8, the 20 *RcTCP* genes exhibited significant tissue-specific expression differences (*P* < 0.05). Among them, the expression levels of the *RcTCP02/10/13/14/15/16/18/19* genes were highest in the roots; the *RcTCP03/04/05/08/09/11/17/20* genes were most expressed in the cotyledons; the *RcTCP01/07/12* genes exhibited the highest expression in the stems; and the *RcTCP06* gene had the highest expression in the true leaves. Eleven *RcTCP* genes showed the lowest expression levels in the stems, including *RcTCP05/06/08/10/11/13/14/15/18/19/20*; six *RcTCP* genes had the lowest expression levels in the true leaves, including *RcTCP01/02/03/04/09/17*; two genes had the lowest expression levels in the cotyledons, including *RcTCP07* and *RcTCP16*; and the *RcTCP12* gene had the lowest expression level in the roots. Among these, the *RcTCP20*, *RcTCP10*, and *RcTCP12* genes exhibited the greatest fold difference in tissue-specific expression, with fold differences between the extremes being 43.44, 41.54, and 21.56, respectively. By contrast, the *RcTCP03*, *RcTCP04*, and *RcTCP06* genes demonstrated the smallest fold difference between tissues, with fold differences between the extremes being 1.52, 2.41, and 3.74, respectively. The results above indicate that the role played by the *RcTCP* gene in different tissues during the 4-leaf stage is varied.

### Analysis of the expression patterns of *RcTCP* genes under low-temperature stress

**Fig. 9:**
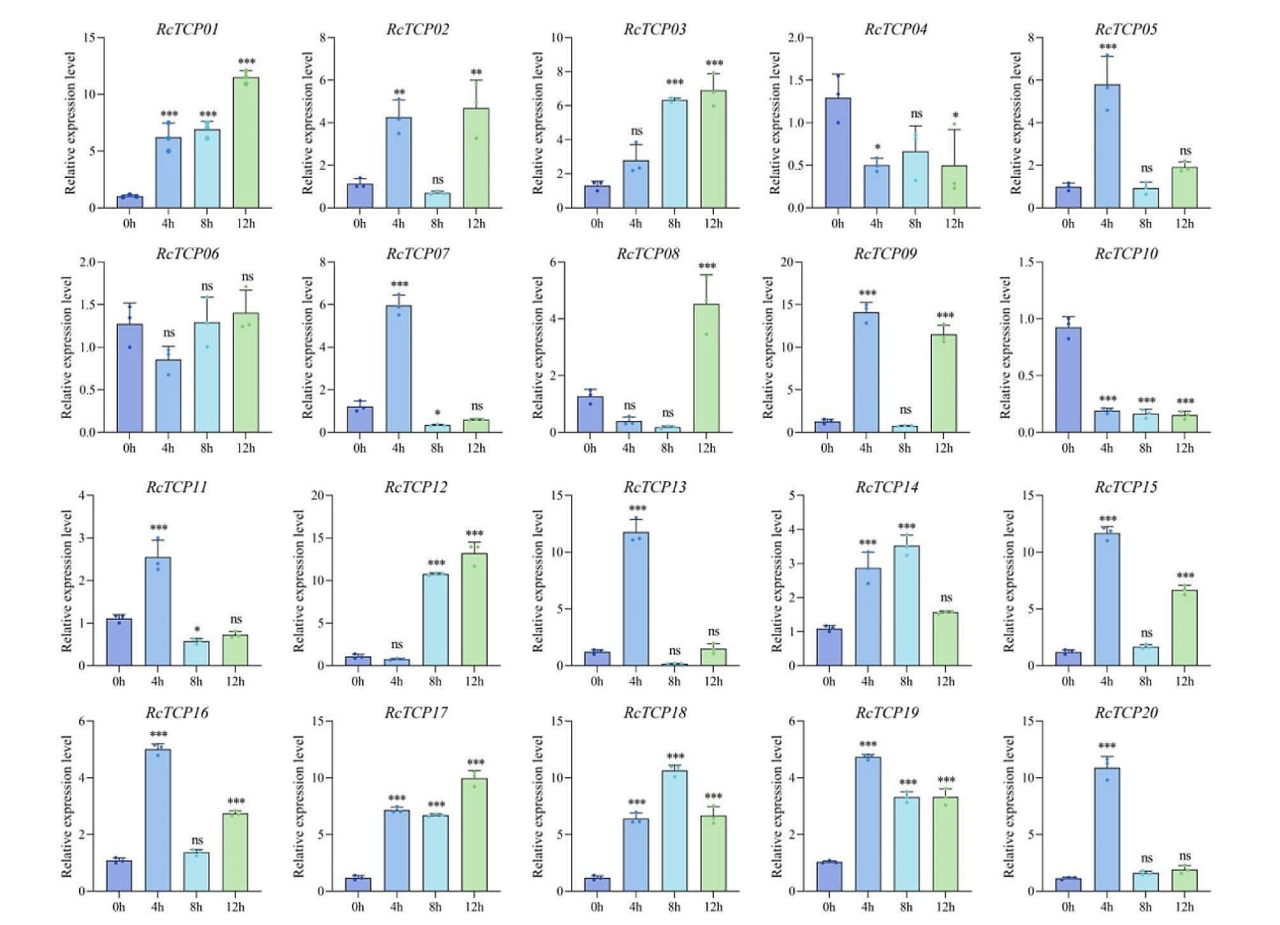
Gene expression of 20 *RcTCP* genes under low-temperature stress at 0 h, 4 h, 8 h and 12 h was analyzed using qRT-PCR. Error bars represent standard errors of three biological replicates. *, ** and *** denote significance at *p* < 0.05, *p* < 0.01, and *p* < 0.001 respectively, compared with 0 h based on Student’s t-test

To clarify the expression patterns of *RcTCP* genes under low-temperature treatment, the relative expression patterns of 20 *RcTCP* genes were analyzed using quantitative real-time (qRT)-PCR. As shown in Fig. 9, with the exception of the *RcTCP06* gene, which showed no significant difference in expression level under low-temperature stress, the expression of the remaining 19 *RcTCP* genes was induced by low-temperature stress. The expression of the *RcTCP01/02/03/05/09/14/15/16/17/18/19/20* genes was activated by low-temperature stress, and their relative expression levels at 4 h, 8 h, and 12 h were higher than at 0 h (control). Among these genes, the expression levels of *RcTCP01* and *RcTCP03* continued to increase; *RcTCP07*, *RcTCP11*, and *RcTCP13* showed the highest expression levels at 4 h, with lower levels at 8 h and 12 h, indicating a pattern of an initial increase followed by a decrease; *RcTCP08* and *RcTCP12* reached their highest expression levels at 12 h, with lower levels detected at 4 h, indicating a pattern of an initial decrease and then an increase. The expression levels of the *RcTCP04* and *RcTCP10* genes continually decreased, indicating that their expression was suppressed at low temperature. Thus, RcTCP01, *RcTCP03*, *RcTCP04*, and *RcTCP10* are key responsive genes of castor bean under low-temperature stress.

### Analysis of the expression patterns of abscisic acid (ABA)-induced *RcTCP* genes

**Fig. 10:**
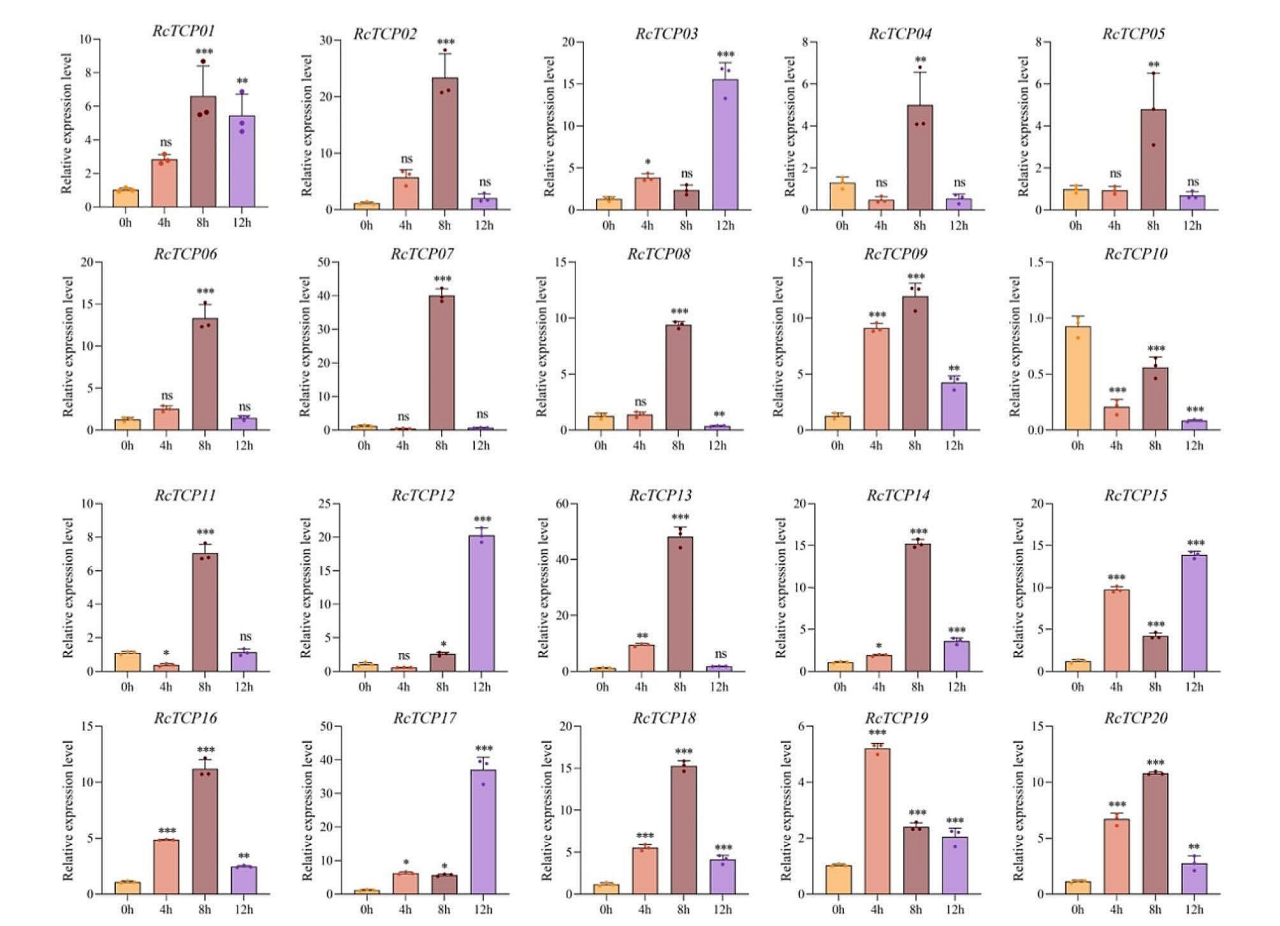
Gene expression of 20 *RcTCP* genes under ABA stress at 0 h, 4 h, 8 h and 12 h was analyzed using qRT-PCR. Error bars represent standard errors of three biological replicates. *, ** and *** denote significance at *p* < 0.05, *p* < 0.01, and *p* < 0.001 respectively, compared with 0 h based on Student’s t-test

Due to the presence of multiple hormone-responsive *cis-*elements in the *RcTCP* promoter region, we analyzed the expression patterns of *RcTCP* genes under ABA stress. As shown in Fig. 10, the expression of the 20 *RcTCP* genes was induced by ABA, with the exception of *RcTCP10*, which was suppressed, while the others were activated. Interestingly, most genes, including *RcTCP01/02/04/05/06/07/08/09/11/13/14/16/18/20*, peaked at 8 h of stress, while *RcTCP03/12/15/17* peaked at 12 h, indicating the delayed expression of *RcTCP* genes under ABA stress. The *RcTCP10* gene may be a key responsive gene in castor bean under ABA stress.

### Analysis of the expression patterns of *RcTCP* genes under drought stress

**Fig. 11:**
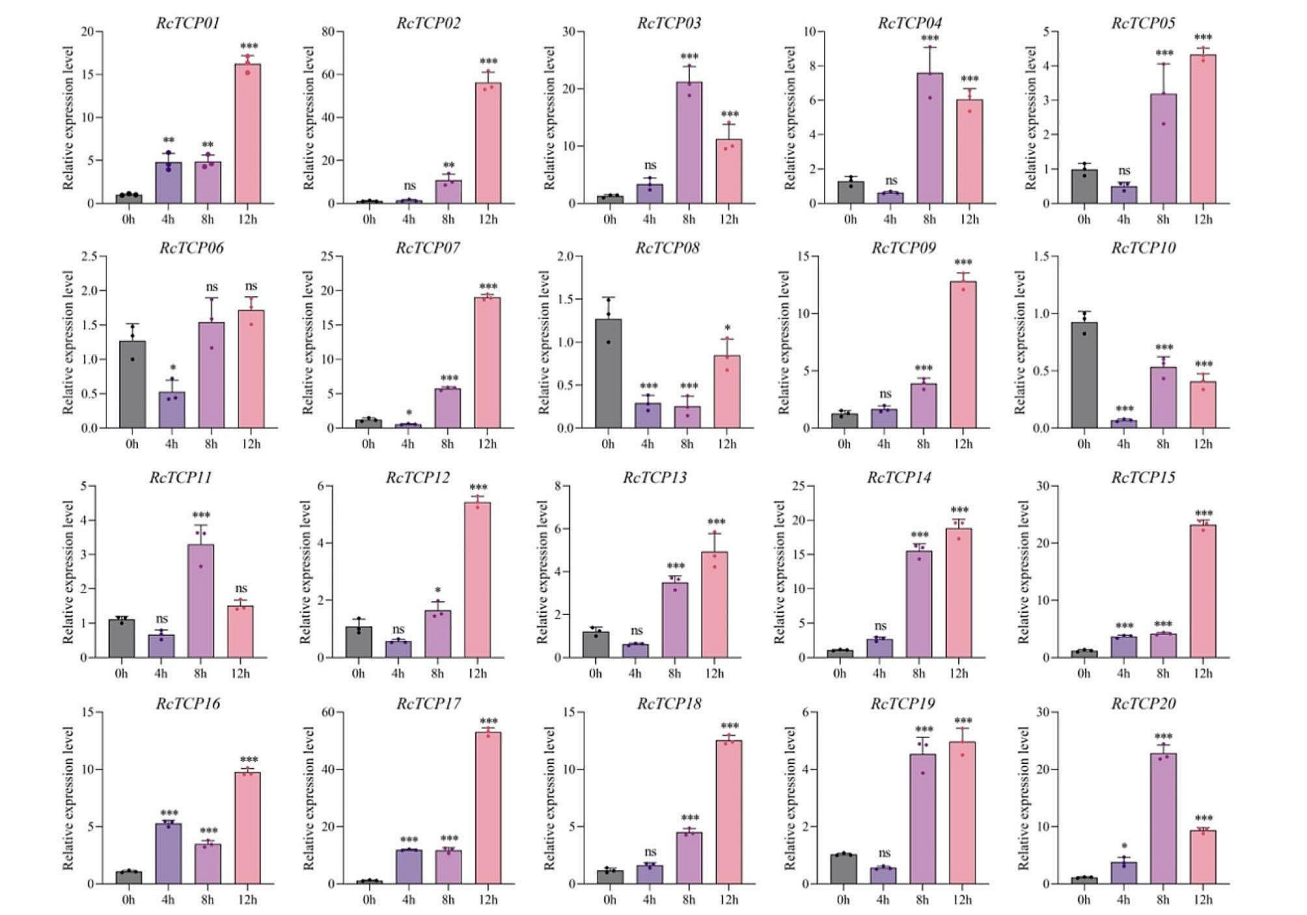
Gene expression of 20 *RcTCP* genes under drought stress at 0 h, 4 h, 8 h and 12 h was analyzed using qRT-PCR. Error bars represent standard errors of three biological replicates. *, ** and *** denote significance at *p* < 0.05, *p* < 0.01, and *p* < 0.001 respectively, compared with 0 h based on Student’s t-test

As shown in Fig. 11, the expression of the *RcTCP08* and *RcTCP10* genes was suppressed under drought stress. The expression values at 4 h, 8 h, and 12 h were all lower than at 0 h. The expression levels of the *RcTCP01/02/09/14/15/17/18* genes continued to increase under stress, indicating activated expression. Interestingly, the expression levels of the *RcTCP04/05/06/07/11/12/13/19* genes were lower at 4 h than at 0 h, and then increased after 4 h, suggesting that these genes exhibited a delayed response to drought stress. Out of the 20 genes, 14 genes showed the highest expression at 12 h, including the *RcTCP01/02/05/06/07/09/12/13/14/15/16/17/18/19* genes, indicating that the *RcTCP* genes in castor bean exhibit a sustained response to drought stress.

### Analysis of the expression patterns of *RcTCP* genes under high-salt stress

**Fig. 12:**
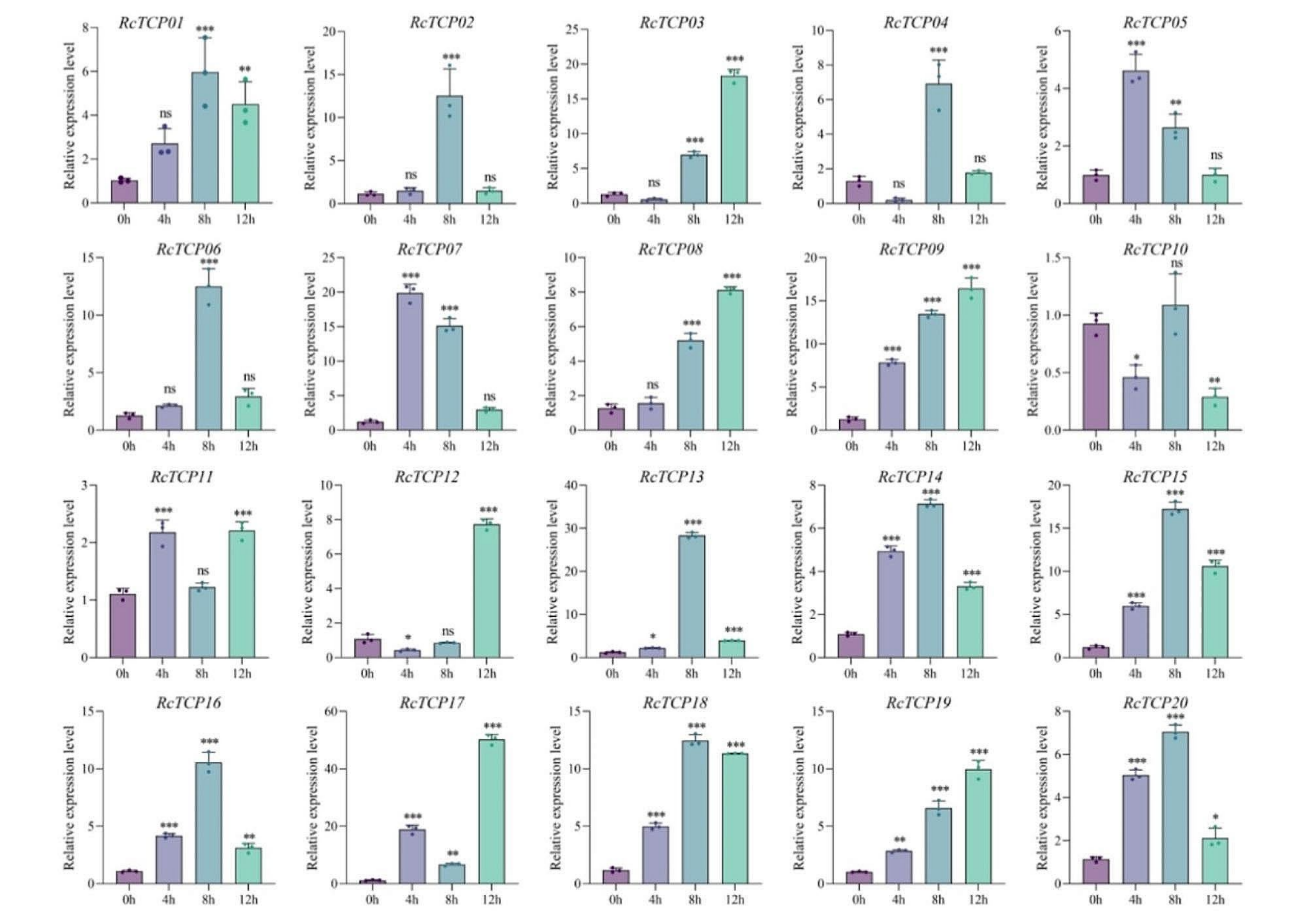
Gene expression of 20 *RcTCP* genes under high salt stress at 0 h, 4 h, 8 h and 12 h was analyzed using qRT-PCR. Error bars represent standard errors of three biological replicates. *, ** and *** denote significance at *p* < 0.05, *p* < 0.01, and *p* < 0.001 respectively, compared with 0 h based on Student’s t-test

The potential functions of *RcTCP* genes under high salt stress were explored. As shown in Fig. 12, the expression levels of the *RcTCP08*, *RcTCP09*, and *RcTCP19* genes were continuously induced under salt stress, showing a gradually increasing trend. The expression of the *RcTCP03*/*04*/*10*/*12* genes was suppressed at 4 h of salt stress, followed by an increase in expression levels. The expression of the *RcTCP01/02/05/06/07/11/13/14/15/16/18/20* genes showed an initial increase followed by a decrease, and the expression levels at 4 h, 8 h, and 12 h were higher than at 0 h. In addition, the expression of 11 *RcTCP* genes peaked at 8 h, including *RcTCP01/02/04/06/10/13/14/15/16/18/20*. Therefore, *RcTCP* genes actively respond to high salt stress.

## Discussion

Currently, *TCP* genes have been identified in many plants, including 24, 22, and 41 genes in *Arabidopsis* [26], rice (*Oryza sativa*) [26], and *Solanum muricatum* [27], respectively. We identified 20 castor bean *RcTCP* genes, further supporting the variability in the number of *TCP* genes among different species. Previous studies have shown that gene duplication contributes to the expansion of gene family members, and segmental duplication enhances gene function through an additive effect [28]. *RcTCP* genes have undergone segmental duplication, resulting in similar gene structures and compositions in deduced protein motifs. Additionally, phylogenetic evolutionary trees constructed for *RcTCP* genes in *Arabidopsis* and rice (*Oryza sativa*) revealed two gene classes, with high homology between *RcTCP* and *AtTCP* genes and significant collinearity. Genes within the same class exhibit functional redundancy [29], suggesting similar biological functions for these homologous genes. Analysis of the selection pressure between genes can significantly contribute to the understanding of gene evolution. *RcTCP* genes exhibit abnormal Ks values compared to *OsTCP* genes, while the Ka/Ks ratio of *AtTCP* genes is less than 1, indicating purifying selection and implying similar functions between *RcTCP* and *AtTCP* genes. For example, the *AtTCP12* gene affects *Arabidopsis* branching development [30], suggesting that the *RcTCP07* gene may be involved in castor bean growth and development. *AtTCP14/15/23* are involved in plant responses to biotic and abiotic stress [30–32], implying potential roles for the *RcTCP03* and *RcTCP05* genes in castor bean stress regulation. Additionally, *AtTCP14* and *AtTCP15* are involved in hormone signaling pathways [30, 31], suggesting that the *RcTCP03*, *RcTCP05*, and *RcTCP01* genes may be involved in hormone signaling transduction. The promoter regions of these three genes all contain ABRE elements, indicating that they may be regulated by ABA hormone. This hypothesis was confirmed by qRT-PCR results.

At the transcriptional level, *TCP* genes can regulate the expression of downstream genes in multiple ways, which also influences their own expression [33, 34]. For example, in rice, the IPA1 (IDEAL PLANT ARCHITECTURE 1) protein has been shown to directly inhibit the transcription of *OsTB1*, thus affecting rice tillering [34]. At the post-transcriptional level, miRNA-mediated mRNA degradation is also an important regulatory mechanism [35]. miR319, as a star target site of *TCP* genes, participates in the transcription of *TCP* genes [35]. For example, in cotton fiber, miR319 serves as a target site for the mRNA of *GhTCP2/3/4/10* genes, regulating the elongation of fiber cells and the thickening of cell walls [36]. miR319a2 reduces the transcription level of Chinese cabbage (*Brassica rapa* ssp.) *BrpTCP2/3/4* genes, affecting the development of cabbage head shape [37]. miR319 targeting is associated with reduced disease resistance in plants with *BrpTCP2/3/10* genes. We predicted four homologous miRNAs of miR319, namely miR319a to miR319d, and all of these miRNAs target the mRNA of *RcTCP* genes, specifically *RcTCP08/12/17/18*. This suggests that *RcTCP08/12/17/18* genes may have similar functions to Class II *AtTCP1/2/3/4* genes and that their transcription is influenced by miR319.

Studies have shown that *TCP* genes do not act alone in the development and stress response of plants, but interact with homologous *TCP* genes or other transcription factors to function together [26–30]. *AtTCP21*, as an important component of the *Arabidopsis* circadian rhythm network, can specifically bind to the promoter region of the *CCA1* (*circadian clock associated 1*) gene, suppressing the expression of the *AtTCP21* gene to mitigate the effect of environmental stress on plants [13, 38]. Related research has also found interactions between AtTCP2/3/11/15 proteins, regulating circadian rhythm in plants [39]. It was found that RcTCP03/06/11 proteins have the most interactions, while AtTCP3/17/7, as homologous proteins of RcTCP, mainly function in *Arabidopsis* cell proliferation and leaf development [40], indicating a possible functional redundancy and influence on castor leaf development. The tissue expression of *RcTCP03/06/11* genes provides strong evidence for this inference.

The expression patterns of genes are important manifestations of their function [41]. Numerous studies have shown that the expression of *TCP* genes is influenced by abiotic stress [42–44]. For example, under salt stress, the *PeTCP10* gene in bamboo is significantly expressed in multiple organs (mature leaves, roots, and stems), and the heterologous expression of the *PeTCP10* gene in *Arabidopsis* enhances salt tolerance [15]. The expression of maize *ZmTCP32* and *ZmTCP42* genes is activated by ABA and polyethylene glycol (PEG) stress, and through the ABA signaling pathway, enhances the drought tolerance of transgenic *Arabidopsis* [43]. The genes *CnTCP2/4/14* in *Chrysanthemum nankingense* are suppressed by low-temperature stress, and overexpression of the *CnTCP2* gene in *Arabidopsis* results in a hypersensitive response to low temperature [44]. The expression of *RcTCP* genes in castor bean is affected by low temperature, ABA, PEG, and salt stress. The *RcTCP01* and *RcTCP03* genes were continuously upregulated under low temperature, and their expression levels in the cotyledons were relatively high, which is consistent with the role of the *AtTCP9* and *AtTCP15* genes in *Arabidopsis* leaves. The *RcTCP04* and *RcTCP10* genes were downregulated under stress conditions, suggesting that they may be negative regulators in castor bean under low-temperature conditions. In addition, the genes *RcTCP09/14/15/18* were continuously activated by PEG, and it is speculated that these genes regulate the impact of drought stress on castor bean in the root and cotyledon tissues. Finally, potential key genes under high salt stress in castor bean, such as *RcTCP08/09/19*, were continuously induced by high salt stress and may regulate plant stress resistance through the ABA pathway. In conclusion, this study found that *RcTCP* genes are likely playing a positive role in the response of castor bean to abiotic stress, providing a foundation for the further analysis of *RcTCP* gene function and mechanisms.

## Conclusion

This study identified 20 members of the *RcTCP* gene family in castor and analyzed their evolutionary relationships with other species, subcellular localizations, and relative expression patterns. All *RcTCP* gene members were found to possess a TCP domain. The RcTC01/02/03/10/16/18 proteins were all localized in the cell nucleus, and *RcTCP* family members possessed multiple miRNA target sites. Interaction was detected between the encoded proteins. In addition, the expression of these genes was induced by various abiotic stresses, and potential functional genes, namely *RcTCP01/03/04/08/09/10/14/15/18/19*, were identified. This study provides a basis for the future exploration of the function of *RcTCP* genes.

## Materials and methods

### Identification and basic information of castor *RcTCP* genes

First, the pfam (PF03634) file of the *TCP* gene family was downloaded from the Pfam (https://pfam.xfam.org) database. Second, we downloaded the castor bean CDS sequence file, genome and annotation files, as well as the protein sequence file from the oil plant database (http://oilplants.iflora.cn), which was generated by Wei et al. [45] assembled the whole genome of wild castor bean ‘Rc039’ (accessions PRJNA706790). The *RcTCP* gene family members of castor were screened using the local HMMER program, and the sequences with missing domains were manually removed. In addition, *Arabidopsis* TCP family proteins were downloaded from the TAIR (https://www.arabidopsis.org) website [46], and the RcTCP proteins of castor were screened using the local Blast program and then integrated with the HMMER results. Finally, a total of 20 *RcTCP* gene family members were screened in castor. The basic physicochemical properties and subcellular locations of the proteins were predicted using the ExPASy (https://www.expasy.org) and WoLF PSORT (https://wolfpsort.hgc.jp/) tools, respectively.

### Evolutionary relationships and gene structure analysis of castor *RcTCP* genes

Phylogenetic trees of the 20 RcTCP proteins in castor were reconstructed and visualized in MEGA 11.0 software. The bootstrap replicates were set to 1000, and the other parameters remained at default values. The 20 protein sequences were submitted to the MEME (https://meme-suite.org/meme/doc/meme.html) website to predict the conserved motifs, with the number of motifs set to 10 and the other parameters kept at default values [47]. The XML files were saved and submitted to Tbtools for visualization. The domain files of the family members were obtained by NCBI-CD Search (https://www.ncbi.nlm.nih.gov/Structure/cdd/wrpsb.cgi) and were manually adjusted and submitted to Tbtools for visualization. Finally, Tbtools [48] was used to display the structural regions of the RcTCP family members, including the CDS region and UTR region, conserved motifs, and domains.

### Secondary and tertiary structure prediction of castor RcTCP proteins

The secondary structure of the RcTCP proteins was predicted by SOPMA (http://npsa-pbil.ibcp.fr/cgi-bin/npsa_automat.pl?page=npsa_sopma.html) [49]. The protein sequences of the *RcTCP* gene family members were submitted to SWISS-MODEL (https://www.swissmodel.expasy.org/) to predict the tertiary structure of the RcTCP proteins [50].

### The position and collinear relationship of castor *RcTCP* genes in the chromosomes

Tbtools was used to extract the location and common relationship information of the *RcTCP* family members in the castor gene annotation files, and Advanced Circos was used to visualize the results.

### Analysis of the systematic evolution and selection pressure of the *TCP* gene family

Integrated the protein sequences of 20 castor bean, 24 *Arabidopsis*, and 22 rice *TCP* genes into one file. A phylogenetic tree was reconstructed using the neighbor-joining method with 1000 bootstrap replicates in MEGA 11.0 software. The tree was edited using the iTol (https://itol.embl.de) website [51]. The homologous gene pairs with the RcTCP gene in castor bean were filtered and plotted using the One Step MCScanX-Super Fast feature in TBtools software. The selection pressure value of the homologous gene pairs was calculated using the Simple Ka/Ks Calculator program.

### Prediction of the *cis*-acting elements of *RcTCP* gene promoters in castor

The 1000-bp promoter sequence upstream of the translation initiation site of the 20 family members was obtained using the gene annotation file of castor and submitted to the PlantCare (http://bioinformatics.psb.ugent.be/webtools/plantcare/html/) website [52]. The interactive information obtained by manual collation only retained the response and regulation components, and the prediction results were visualized using Tbtools and Excel software.

### Analyzing PPIs of RcTCP and predicting miRNA target sites in the coding genes

The STRING website (https://cn.string-db.org/) was used to predict the interaction between RcTCP proteins, and Cytoscape 3.7.4 software was used to construct a network of interactions between *RcTCP* genes [53]. The online website psRNATarget (http://plantgrn.noble.org/psRNATarget/) was used to predict miRNAs related to *RcTCP*, and the miRNA network was visualized using the OmicShare tool (http://www.omicshare.com/tools) [54].

### Subcellular localization analysis of RcTCP proteins

The subcellular localization of the RcTCP01/02/03/10/16/18 proteins was determined using specific primers designed without stop codon (Table S4, Fig. S1). To construct the subcellular localization vector 35 S::RcTCP-GFP, the fragments were subcloned into the pCAMBIA2300-GFP vector (35 S::GFP) using the Hieff Clone® One Step Cloning Kit (Yeasen, Shenyang, China). *Arabidopsis* protoplasts were isolated using the Plant Protoplasts Isolation Kit (Beyotime, Shenyang, China), and the empty vector and fusion expression vector were separately transformed into the protoplasts according to the instructions of the Plant Protoplasts Transfection Kit (Beyotime, Shenyang, China). Then, the protoplasts, cultured at 22 °C in a growth chamber for 16 h, were observed using a confocal laser scanning microscope.

### Castor material processing and expression pattern analysis of *RcTCP* genes

‘Tongbi No.5’ was used as the experimental material was generously provided by the Tongliao Academy of Agriculture Sciences, China. *Ricinus* seeds were sterilized with 75% alcohol for 30 s and then germinated at 28 °C. After 4 days, uniformly germinated seeds were selected and placed in three germination boxes measuring 32 × 25.5 × 12 cm. The seedlings were cultured in an incubator at 25 °C with 16 h of light (light intensity 450 µmol m^− 2^s^− 1^) and 70–75% relative humidity. Once the two cotyledons had opened, the plants were inundated with 1/2 Hoagland’s nutrient solution. Seedlings at the 4-leaf stage were subjected to low temperature (4 °C) stress, ABA (150 µmol·L^− 1^) stress, drought (10% PEG6000 simulated drought) stress, and high salt (300 mmol·L^− 1^) stress. Tissues (roots, stems, cotyledons, and true leaves) were harvested at time points of 0 h, 4 h, 8 h, and 12 h, and each treatment was repeated three times. Total RNA was extracted from the plants using the Total RNA Extractor (Sangon) Kit (Fig. S2), and the RNA concentration and purity were detected on a Qubit2.0 fluorometer (Invitrogen). The PrimeScript™ RT reagent Kit (Perfect Real Time) (Takara, Shanghai, China) was used to remove genomic DNA, and qRT-PCR reactions were performed on a qTOWER^3^G (Analytik Jena, Germany) instrument using 2XRealStar Fast SYBR gPCR Mix (Genstar, Shenyang, China) according to the manufacturer’s instructions. Each reaction consisted of three technical replicates, using *actin* as the reference gene, and primers were designed using Snap Gene 4.3.6 software (Table S1). The relative expression levels of genes at different time points were calculated using the 2^−ΔΔCT^ method, and graphs were generated using TBtools and GraphPad Prism 9.5.1 software.

### Electronic supplementary material

Below is the link to the electronic supplementary material.

Supplementary Material 1

## Data Availability

The datasets used and/or analysed during the current study available from the corresponding author on reasonable request.
