# Supplementary material for: Identification and characterization of the RcTCP gene family and its expression in response to abiotic stresses in castor bean
Source: BMC Genomics. 2024 Jul 4;25:670. doi: 10.1186/s12864-024-10347-6 (PMC11223397; doi:10.1186/s12864-024-10347-6)
Supplement: Supplementary file 1 — Supplementary Material 1 [file 12864_2024_10347_MOESM1_ESM.docx]

**Table S1** Primer used in real-time quantitative PCR.

| *Actin*-F | 5’-GTCGACCAGGGAGGAGT-3’ |
| --- | --- |
| *Actin*-R | 5’-CCACATCCACAGGAACCATT-3’ |
| *RcTCP01*-F | 5’-GCGAGTACATTGACATAACCGACAC-3’ |
| *RcTCP01*-R | 5’-ACCCTGAGGAACTACTGCTTGTTG-3’ |
| *RcTCP02*-F | 5’-AAGAATCAACGATGCCGACAATGAG-3’ |
| *RcTCP02*-R | 5’-TTGGAAACACATGCTGCTGGTTAC-3’ |
| *RcTCP03*-F | 5’-AGCAGCAACAGCAACAACTTATCTC-3’ |
| *RcTCP03*-R | 5’-TTAGTGTGACGGTCTTTGGTGGAG-3’ |
| *RcTCP04*-F | 5’-CACCGACAACGACACAACAAGAAG-3’ |
| *RcTCP04*-R | 5’-TGGTGTGGCAAGAATGTTTGATGG-3’ |
| *RcTCP05*-F | 5’-CACCACCACCACCACCATCAC-3’ |
| *RcTCP05*-R | 5’-AAGGAGAGGAGGAGGAGCAAGG-3’ |
| *RcTCP06*-F | 5’-CAGCTTTCTCCTCGGGTTCTCAG-3’ |
| *RcTCP06*-R | 5’-TGTTTCCCTCGGCTCATTATTCTCC-3’ |
| *RcTCP07*-F | 5’-ACGGAGGGTGACTCATTTGTAAGAC-3’ |
| *RcTCP07*-R | 5’-CTTGCTCTTGCTCTTGCCTTGTC-3’ |
| *RcTCP08*-F | 5’-TGCTGCTGCTGCTGCTACTAG-3’ |
| *RcTCP08*-R | 5’-AGCCCTGTGCCTCCAAAGTAATC-3’ |
| *RcTCP09*-F | 5’-CGTGTGGAGGTGGTGAGATAGAAG-3’ |
| *RcTCP09*-R | 5’-AGCAGTAGAAGCAGCAGCTATCC-3’ |
| *RcTCP10*-F | 5’-CAGCCTAGCAAGGTTGTTGATTGG-3’ |
| *RcTCP10*-R | 5’-AATTCATTGGTGGCATTGGAAGTGG-3’ |
| *RcTCP11*-F | 5’-AACGACGGAGGAGGATGAGAATAAC-3’ |
| *RcTCP11*-R | 5’-CTTGATGAGGCTGCGGAGGAG-3’ |
| *RcTCP12*-F | 5’-GCCAACGCCAACACCAACAG-3’ |
| *RcTCP12*-R | 5’-CTTGAACCTCCACTATCTCGCCTAC-3’ |
| *RcTCP13*-F | 5’-AATCCTCCTGCTCCTGCTAACATAC-3’ |
| *RcTCP13*-R | 5’-ATCATCAGCCCTTCTCTTCCTTCAC-3’ |
| *RcTCP14*-F | 5’-TTGGGTTTCCTGGGTTTGATTTGC-3’ |
| *RcTCP14*-R | 5’-CAGGTATCTGCTGGTTGCTAGTCC-3’ |
| *RcTCP15*-F | 5’-CAGGTATCTGCTGGTTGCTAGTCC-3’ |
| *RcTCP15*-R | 5’-CTGCTGCTGCTGCTGTTGATG-3’ |
| *RcTCP16*-F | 5’-CGGGTCAAGAGATAGGGCTAACTG-3’ |
| *RcTCP16*-R | 5’-GCTCCGATTCCACCGACAGAAG-3’ |
| *RcTCP17*-F | 5’-GAAGCATTGGCAGGTCATAGCATTC-3’ |
| *RcTCP17*-R | 5’-TTGGAGAAGCAGAGGAAGGCATATC-3’ |
| *RcTCP18*-F | 5’-TCACCAACAACATCCTCCACTTCTC-3’ |
| *RcTCP18*-R | 5’-TCCACTGCCTTTCATGCCTCTTC-3’ |
| *RcTCP19*-F | 5’-TCACAACCCAGAACCCAACTCAC-3’ |
| *RcTCP19*-R | 5’-GAAAGATCCGAGCAGCACAAAGAG-3’ |
| *RcTCP20*-F | 5’-TCCTGGAGGTAGCGTCATTGTTTC-3’ |
| *RcTCP20*-R | 5’-TCCCAACCGTGCTAAATCCTGTC-3’ |

| Gene 1 | Gene 1 position | Gene 2 | Gene 2 position | Ka | Ks | Ka/Ks | Duplication type | Note |
| --- | --- | --- | --- | --- | --- | --- | --- | --- |
| *RcTCP04* | 10067956-10070293 | *AtTCP23* | 13115826-13117235 | 0.34 | 2.22 | 0.15 | Segmental |  |
| *RcTCP05* | 25614693-25616449 | *AtTCP15* | 26216009-26218071 | 0.39 | 4.61 | 0.09 | Segmental |  |
| *RcTCP05* | 25614693-25616449 | *AtTCP14* | 17558793-17561259 | 0.34 | 3.06 | 0.11 | Segmental |  |
| *RcTCP09* | 17943131-17948623 | *AtTCP1* | 25168228-25169307 | 0.51 | 3.08 | 0.16 | Segmental |  |
| *RcTCP03* | 4289408-4291849 | *AtTCP15* | 26216009-26218071 | 0.41 | 2.15 | 0.19 | Segmental |  |
| *RcTCP07* | 23517288-23519271 | *AtTCP12* | 25168228-25169307 | 0.56 | 2.57 | 0.22 | Segmental |  |
| *RcTCP18* | 1748223-1751113 | *AtTCP10* | 13220478-13222609 | 0.36 | NaN | NaN | Segmental | High Sequence Divergence Value (pS>=0.75) |
| *RcTCP04* | 10067956-10070293 | *AtTCP22* | 13115826-13117235 | 0.40 | NaN | NaN | Segmental | High Sequence Divergence Value (pS>=0.75) |
| *RcTCP15* | 15990001-15992857 | *AtTCP8* | 21512459-21514022 | 0.35 | 1.83 | 0.19 | Segmental |  |
| *RcTCP01* | 1491422-1493654 | *AtTCP9* | 18820242-18821889 | 0.25 | NaN | NaN | Segmental | High Sequence Divergence Value (pS>=0.75) |
| *RcTCP01* | 1491422-1493654 | *AtTCP19* | 18820242-18821889 | 0.41 | NaN | NaN | Segmental | High Sequence Divergence Value (pS>=0.75) |
| *RcTCP14* | 12527419-12530453 | *AtTCP20* | 9957376-9958945 | 0.28 | 2.08 | 0.13 | Segmental |  |
| *RcTCP14* | 12527419-12530453 | *AtTCP6* | 9957810-9958754 | 0.40 | NaN | NaN | Segmental | High Sequence Divergence Value (pS>=0.75) |
| *RcTCP19* | 14776301-14777380 | *AtTCP11* | 15540246-15541248 | 0.38 | NaN | NaN | Segmental | High Sequence Divergence Value (pS>=0.75) |
| *RcTCP10* | 9398670-9401202 | *AtTCP13* | 390720-392767 | 0.50 | NaN | NaN | Segmental | High Sequence Divergence Value (pS>=0.75) |
| *RcTCP10* | 9398670-9401202 | *AtTCP5* | 391522-392589 | 0.58 | NaN | NaN | Segmental | High Sequence Divergence Value (pS>=0.75) |
| *RcTCP05* | 25614693-25616449 | *OsTCP6* | 31398595-31397363 | 1.18 | NaN | NaN | Segmental | High Sequence Divergence Value (pS>=0.75) |
| *RcTCP03* | 4289408-4291849 | *OsTCP6* | 31398595-31397363 | 1.20 | NaN | NaN | Segmental | High Sequence Divergence Value (pS>=0.75) |
| *RcTCP03* | 4289408-4291849 | *OsTCP12* | 6572477-6571314 | 0.43 | NaN | NaN | Segmental | High Sequence Divergence Value (pS>=0.75) |
| *RcTCP01* | 1491422-1493654 | *OsTCP18* | 20365094-20363949 | 0.65 | NaN | NaN | Segmental | High Sequence Divergence Value (pS>=0.75) |
| *RcTCP02* | 10067956-10070293 | *RcTCP07* | 23517288-23519271 | 0.59 | 2.86 | 0.21 | Segmental |  |
| *RcTCP03* | 4289408-4291849 | *RcTCP05* | 25614693-25616449 | 0.32 | 1.76 | 0.18 | Segmental |  |
| *RcTCP06* | 7816619-7819252 | *RcTCP10* | 9398670-9401202 | 0.40 | 0.99 | 0.41 | Segmental |  |
| *RcTCP13* | 2019034-2020663 | *RcTCP19* | 14776301-14777380 | 0.55 | 1.72 | 0.32 | Segmental |  |

**Table S2** Analysis of selection pressure on co-linear gene pairs. NaN indicates uncalculatable values, indicating large genetic distance.

**Table S3** Prediction of the secondary structure of RcTCP protein.

| Gene Name | Alpha helix | Extended strand | β-turn | Random coil |
| --- | --- | --- | --- | --- |
| RcTCP01 | 18.77% | 21.41% | 0 | 59.82% |
| RcTCP02 | 30.22% | 16.79% | 0 | 53.00% |
| RcTCP03 | 28.57% | 16.43% | 0 | 55.00% |
| RcTCP04 | 26.98% | 14.97% | 0 | 58.05% |
| RcTCP05 | 25.44% | 12.97% | 0 | 61.60% |
| RcTCP06 | 15.00% | 18.33% | 0 | 66.67% |
| RcTCP07 | 32.74% | 16.70% | 0 | 50.56% |
| RcTCP08 | 27.16% | 9.66% | 0 | 63.18% |
| RcTCP09 | 34.26% | 21.32% | 0 | 44.42% |
| RcTCP10 | 25.88% | 11.33% | 0 | 62.79% |
| RcTCP11 | 22.71% | 20.52% | 0 | 56.77% |
| RcTCP12 | 33.69% | 13.40% | 0 | 52.91% |
| RcTCP13 | 15.23% | 12.69% | 0 | 72.08% |
| RcTCP14 | 32.85% | 17.15% | 0 | 50.00% |
| RcTCP15 | 27.84% | 14.10% | 0 | 58.06% |
| RcTCP16 | 27.84% | 14.10% | 0 | 58.06% |
| RcTCP17 | 32.27% | 8.80% | 0 | 58.92% |
| RcTCP18 | 31.37% | 10.14% | 0 | 58.49% |
| RcTCP19 | 37.84% | 15.14% | 0 | 47.03% |
| RcTCP20 | 25.11% | 20.59% | 0 | 54.30% |

**Table S4** Primer for *RcTCP* gene cloning

| RcTCP01-F | 5’-ATGGCAACCTTCCATAAAAAGGAAC-3’ |
| --- | --- |
| RcTCP01-R | 5’-ATGCTTCGAAGATCGAGTCATAAAC-3’ |
| RcTCP02-F | 5’-ATGTTTCAGTCAAGCAACAATGGCA-3’ |
| RcTCP02-R | 5’-ACTGTTATAGGCCACTTCCCATGAC-3’ |
| RcTCP03-F | 5’-ATGTTTCAGTCAAGCAACAATGGCA-3’ |
| RcTCP03-R | 5’-GCTAGTTGTATCATGCTCATCCTTT-3’ |
| RcTCP10-F | 5’-ATGATTAATAATTCAAAAGAGGAGG-3’ |
| RcTCP10-R | 5’-CTGCTGATCTTTATTATTATTATTA-3’ |
| RcTCP16-F | 5’-ATGGAAACAAAGGGCCCAAATAATC-3’ |
| RcTCP16-R | 5’-TTGCCTTGATCCTTGAGAATCATCC-3’ |
| RcTCP18-F | 5’-ATGGTGTTATTGGGGTATGGAATCT-3’ |
| RcTCP18-R | 5’-TTCGCCATGTTTGGCTTTGATTCTT-3’ |

**Figure S1.** Cloning of *RcTCP* gene and E. coli liquid PCR. (a) Cloning of *RcTCP* gene. (b) Liquid PCR. In the figure, M represents 2000 bp DNA marker, and 1 to 6 are amplification products of *RcTCP01*, *RcTCP02*, *RcTCP03*, *RcTCP10*, *RcTCP16*, and *RcTCP18* genes, respectively.

**
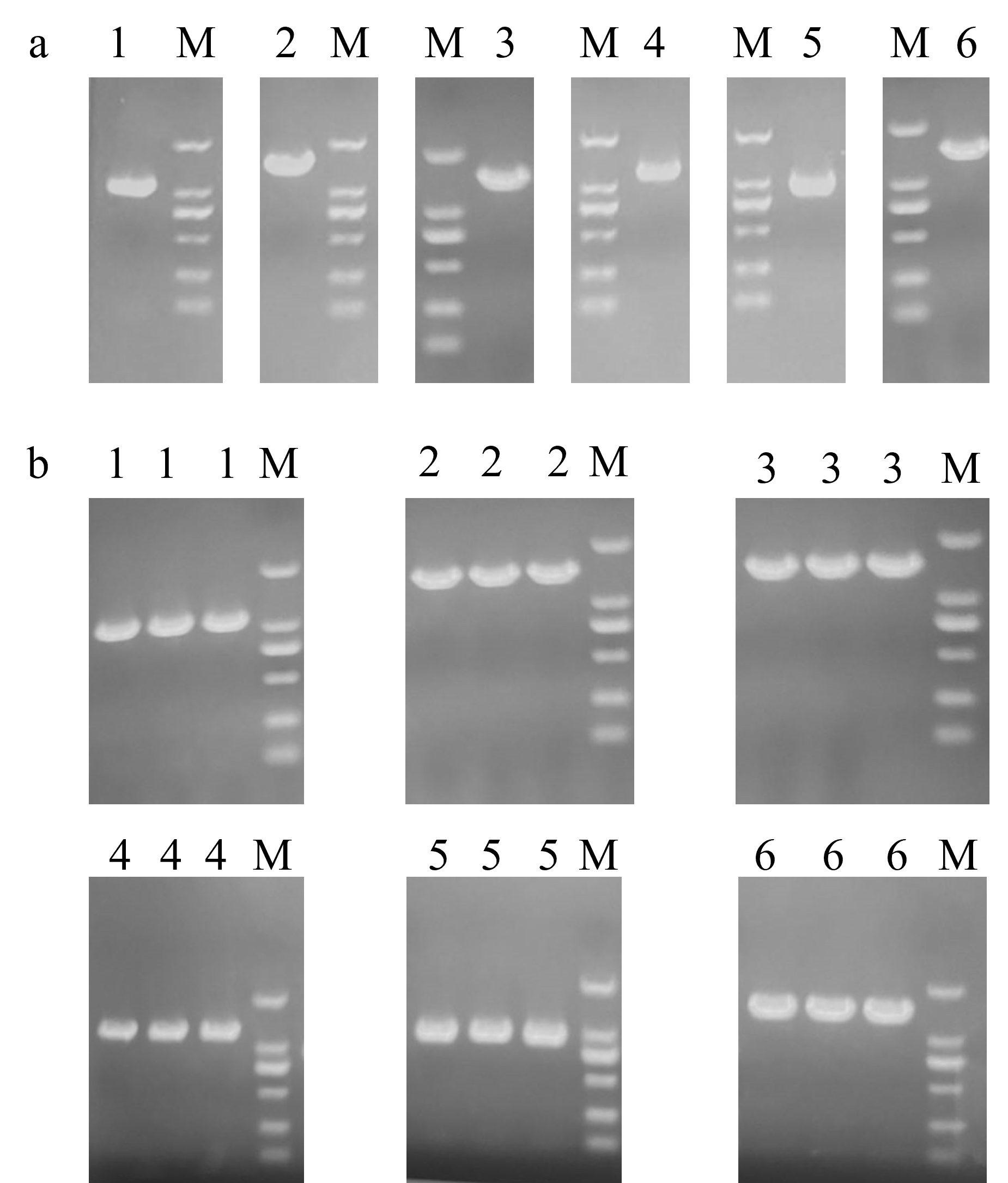
**

**Figure S2.** RNA quality detection. 1-4 were untreated roots, stems, cotyledons and true leaves, respectively. The true leaves of 5-7 were treated with low temperature stress for 4 h, 8 h and 12 h, respectively. The true leaves of 8-10 were treated with ABA stress for 4 h, 8 h and 12 h, respectively. 11-13 were true leaves treated with drought stress for 4 h, 8 h and 12 h, respectively. The true leaves of 14-16 were treated with high salt stress for 4 h, 8 h and 12 h, respectively. The 5S, 18S and 28S fragments of RNA are clearly visible, indicating that the extracted RNA structure is complete. From top to bottom is the repeated group.

**
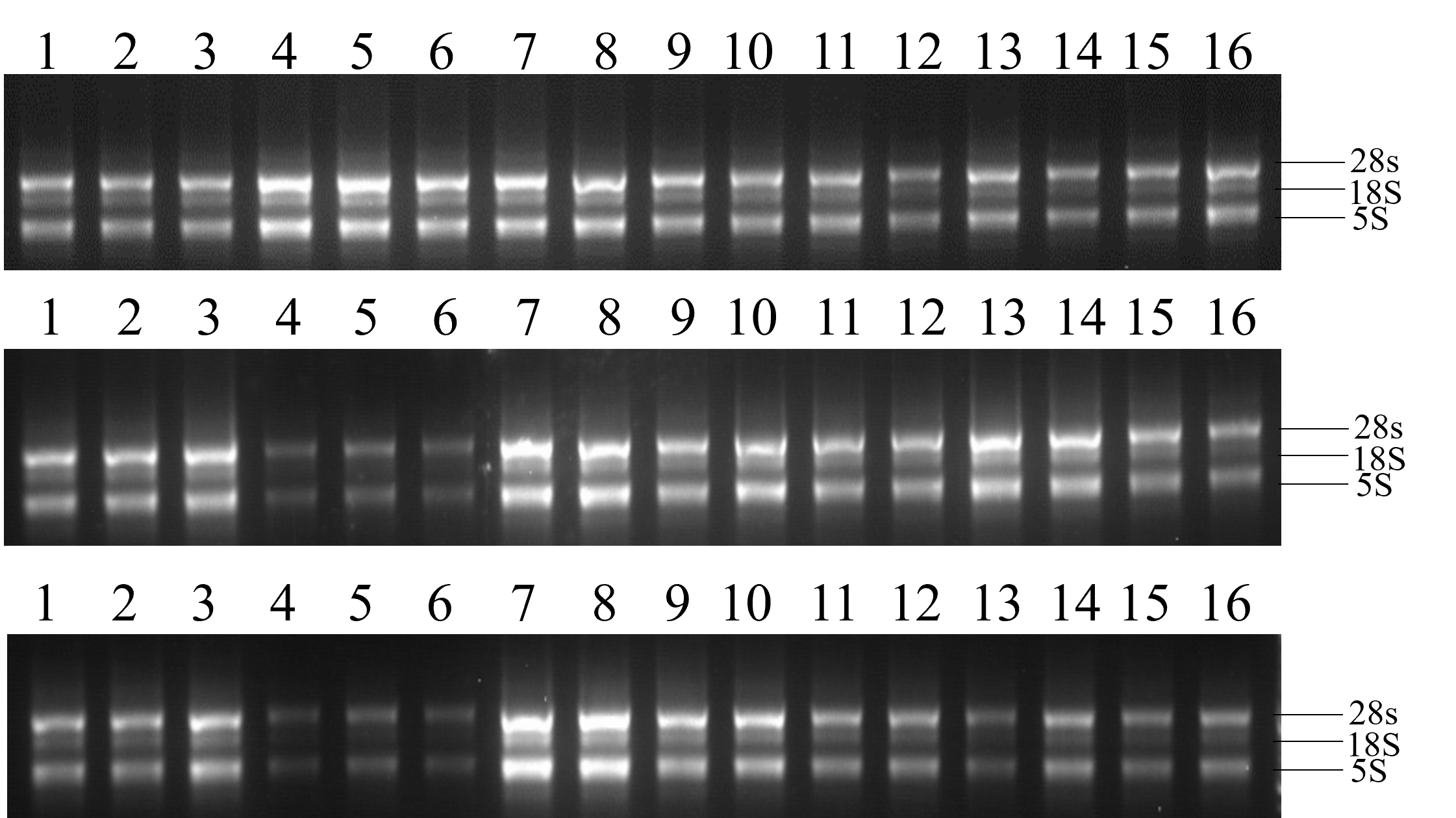
**
